# Encapsulation into sterically stabilised liposomes enhances the immunogenicity of melanoma-associated Melan-A/MART-1 epitopes

**DOI:** 10.1038/sj.bjc.6601473

**Published:** 2004-01-06

**Authors:** M Adamina, M Bolli, F Albo, A Cavazza, P Zajac, E Padovan, R Schumacher, A Reschner, C Feder, W R Marti, D Oertli, M Heberer, G C Spagnoli

**Affiliations:** 1 Department of Surgery, Division of Surgical Research, University of Basel, ZLF, Lab. 401, Hebelstrasse 20, Basel 4031, Switzerland; 2 Department of Neurosciences, University of Rome ‘Tor Vergata’, Rome, Italy

**Keywords:** sterically stabilised liposomes, tumour-associated antigens, active specific immunotherapy, melanoma

## Abstract

Tumour-associated antigens (TAA)-specific vaccination requires highly immunogenic reagents capable of inducing cytotoxic T cells (CTL). Soluble peptides are currently used in clinical applications despite an acknowledged poor immunogenicity. Encapsulation into liposomes has been suggested to improve the immunogenicity of discrete antigen formulations. We comparatively evaluated the capacity of HLA-A2.1 restricted Melan-A/MART-1 epitopes in soluble form (S) or following inclusion into sterically stabilised liposomes (SSL) to be recognised by specific CTL, to stimulate their proliferation and to induce them in healthy donors' peripheral blood mononuclear cells (PBMC), as well as in melanoma-derived tumour-infiltrating lymphocytes (TIL). HLA-A2.1^+^, Melan-A/MART-1-NA-8 melanoma cells served as targets of specific CTL in 51Cr release assays upon pulsing by untreated or human plasma-treated soluble or SSL-encapsulated Melan-A/MART-1 27–35 (M27–35) or 26–35 (M26–35) epitopes. These reagents were also used to stimulate CTL proliferation, measured as 3H-thymidine incorporation, in the presence of immature dendritic cells (iDC), as antigen-presenting cells (APC). Induction of specific CTL upon stimulation with soluble or SSL-encapsulated peptides was attempted in healthy donors' PBMC or melanoma-derived TIL, and monitored by 51Cr release assays and tetramer staining. Na-8 cells pulsing with SSL M27–35 resulted in a five-fold more effective killing by specific CTL as compared with equal amounts of S M27–35. Encapsulation into SSL also provided a partial (50%) protection of M27–35 from plasma hydrolysis. No specific advantages regarding M26–35 were detectable in these assays. However, at low epitope concentrations (⩽100 ng ml^−1^), SSL M26–35 was significantly more effective in inducing CTL proliferation than S M26–35, in the presence of iDC, as APC. Preincubation with iDC for 6 h virtually abolished the capacity of S M26–35 to stimulate specific CTL proliferation, but only partially affected that of SSL M26–35. Most importantly, SSL M26–35 was able to enhance the induction of specific CTL in healthy donors PBMC and in melanoma-derived TIL as compared to S M26–35. Taken together, our data indicate that encapsulation of TAA epitopes into SSL results in effective immunogenic formulations suitable for clinical use in active specific tumour immunotherapy.

The identification of a large series of human tumour-associated antigens (TAA) in the past decade suggests that, upon appropriate immunisation, cellular responses capable of targeting neoplastic cells could be induced (Boon and van der Bruggen, 1996; Renkvist *et al*, 2001). Although the ‘*in vivo*’ generation of effective HLA-class I-restricted cytotoxic T-cell (CTL) responses still represents a challenge, clinical trials have promptly been designed, taking advantage of different immunogenic formulations.

Synthetic peptides have been used in the presence or absence of cytokines or adjuvants in a number of protocols, and their administration has been shown to result in clinical responses (Jaeger *et al*, 1996; Rosenberg *et al*, 1998; Marchand *et al*, 1999). Still, peptides are poor immunogens, characterised by short half-lives ‘*in vitro*’ and ‘*in vivo*’, possibly due to the effects of serum and cellular peptidases (Amoscato *et al*, 1998; Brinckerhoff *et al*, 1999; Blanchet *et al*, 2001; Albo *et al*, 2003), and long immunisation courses are required before specific responsiveness eventually becomes evident (Jaeger *et al*, 1996; Marchand *et al*, 1999, Coulie and van der Bruggen, 2003).

Liposomes constructed according to different formulations have long been used as drug carriers (Langer, 1998; Lasic, 1998) or vaccine components (Alving *et al*, 1995; Gluck, 1995; Gregoriadis, 1999; Gregoriadis *et al*, 1999). Interestingly, liposomes containing lipopeptides derived from MUC1 human TAA and monophosphoryl lipid A as adjuvant were shown to be able to induce specific immune responses *in vitro* (Agrawal *et al*, 1998).

Sterically stabilised liposomes (SSL, Woodle and Lasic, 1992; Allen, 1994) characterised by PEG coating display prolonged bioavailability, but decreased cellular uptake, as compared to conventional liposomes. Interestingly, SSL containing a model antigenic protein have been shown to induce both class I and class II restricted immune responses in mice (Ignatius *et al*, 2000) upon presentation by professional antigen-presenting cells (APC) such as dendritic cells (DC).

We constructed SSL containing immunodominant HLA-A2.1 restricted CTL epitopes derived from Melan-A/MART-1 melanoma TAA (Kawakami *et al*, 1994; Valmori *et al*, 1998), and we have analysed their immunogenicity *in vitro* in comparison with their soluble counterpart. We show here that antigenic peptides encapsulated in SSL display a significantly higher capacity to resist plasma enzymatic hydrolysis, stimulate T-cell proliferation, and to induce specific cytotoxic T-cell responses than their soluble counterparts.

## MATERIALS AND METHODS

### Synthetic peptides

Antigenic Melan-A/MART-1 and control (HLA-A2.1 binding, p21 ras derived, Juretic *et al*, 1996) peptides were produced on a 9050 Millipore peptide synthesiser (Millipore, Volketswil, CH, Switzerland) using *N*-alpha-fluorenylmethoxycarbonylamino-acid pentafluorophenyl esters (Juretic *et al*, 1996). The purity of the reagents thus produced (>95%) was verified by mass spectrometry (courtesy of Dr S Stevanovic, Tübingen, Germany).

### Liposome production and characterisation

Sterically stabilised liposomes were generated upon solubilisation in chloroform (HPLC grade, Fluka, Buchs, CH, Switzerland) of cholesterol, distearylphosphatidylcholine and distearylphosphatidylethanolamine-PEG^2000^ (all synthetic lipids from Genzyme Pharmaceuticals, Liestal, CH, Switzerland). The molar ratio used was 1 : 0.65 phospholipids to cholesterol. The lipids were then dried down in a rotatory evaporator to a thin lipid film. Upon rehydration and mechanical dispersion into a solution of Mart-1/Melan-A_26–35_ peptide in PBS (Life Technologies, Paisley, UK), multilamellar liposomes were formed. Sterically stabilised liposomes batches were then submitted to five cycles of freeze–thaw to obtain a homogenous suspension and optimise peptide encapsulation. Sizing down of SSL to 100 nm small unilamellar vesicles was achieved by using a LiposoFast extruder (Avestin Inc., Ottawa, Canada).

Separation of SSL-encapsulated and soluble peptides was obtained by gel chromatography on Sepharose CL4b columns (Amersham Pharmacia Biotech, Dübendorf, CH, Switzerland) and extensive dialysis. Finally, SSL suspensions were filtered for sterility with a 200 nm filter (Orange Scientific, Waterloo, Belgium). Sterically stabilised liposomes SSL preparations were stored at 4°C in the dark to prevent lipid oxidation.

The size of liposomes in individual SSL batches was tested by photon correlation spectroscopy using a DynaPro-801 molecular sizing instrument equipped with a temperature-controlled microsampler (Protein Solutions, Charlotteville, SC, USA). The unilamellarity of the liposomes was verified by electron microscopy (negative staining technique). Quantification of SSL-encapsulated peptides was performed by fluorescamine assay (Udenfriend *et al*, 1972): fluorescence was measured at 405 nm absorbance and 475 nm emission wavelengths. No fluorescence was recorded on empty SSL or on phospholipids in solution.

SSL preparations were negative (<10 pg ml^−1^) when tested for endotoxin contamination by chromogenic Limulus Amebocyte lysate assay (Charles River, Charleston, SC, USA).

### Cellular reagents and culture conditions

Healthy donors' or patients' PBMC were obtained from heparinised venous blood samples or buffy coat preparations (Blutspendezentrum, Basel, Switzerland) upon Ficoll (Lymphoprep, Nycomed, Oslo, Norway) centrifugation followed by two washes with PBS. Cells were cultured in RPMI1640 medium supplemented with Ciproxin (Bayer, Zurich, Switzerland, 10 *μ*g ml^−1^), glutamine (2 mM), sodium pyruvate, nonessential amino acids, HEPES buffer (GIBCO, Paisley, UK all 1 mM) and 5% heat-inactivated human AB serum, thereafter referred to as complete AB medium. Cytotoxic T-cells clones were generated from melanoma patients' PBMC, as previously detailed (Noppen *et al*, 2000), maintained in culture in complete medium supplemented with 200 U ml^−1^ of recombinant IL-2 (rIL-2, Hoffman-LaRoche, Basel, Switzerland) and periodically re-stimulated with purified PHA (1 *μ*g ml^−1^, Abbott, Baar, Switzerland) in the presence of irradiated allogenic PBMC.

CD14^+^ cells were isolated with magnetic beads (Miltenyi, Bergisch-Gladbach, Germany). Immature dendritic cells were prepared as previously outlined (Remmel *et al*, 2001) from positively sorted CD14^+^ cells upon 5–7 days culture in RPMI 1640 supplemented with 10% FCS, ciproxin, glutamine, sodium pyruvate, nonessential amino acids and HEPES buffer (complete FCS medium), in the presence of GM-CSF (50 ng ml^−1^, Novartis, Basel, CH, Switzerland) and IL-4 (1000 U ml^−1^, courtesy of Dr Lanzavecchia, Bellinzona, CH, Switzerland). These iDC displayed the typical CD14^−^, CD1a^+^ phenotype.

### Functional assays

Cytotoxic activity was tested against Cr^51^-labelled and peptide-pulsed Na-8 cells (courtesy of Dr F Jotereau, Nantes, France) at 1000 cells well^−1^, in the presence of a 100-fold excess of unlabelled K562 cells to quench the background NK-like activity. For each culture, cytotoxicity assays were performed by using, as targets, cells pulsed in the presence of specific or control peptides or SSL preparations. Percent killing was calculated according to the standard formula.

Proliferation of MART-1/Melan-A-specific HLA-A2.1-restricted CTL clones was tested as follows. Cells (1 × 10^5^ in 100 *μ*l of complete AB medium) were seeded in triplicates in flat-bottom, 96-well plates. As APC, 5 × 10^4^ purified, HLA-A2.1-matched, iDC cells were added. Antigenic preparations were then supplemented at the concentrations indicated. In dedicated experiments, APC were preincubated for varying time lengths in the presence of immunogenic preparations and CTL were subsequently added. All cultures were prolonged for 3 days, including an overnight incubation in the presence of ^3^H-thymidine (1 *μ*Ci well^−1^, Amersham, Little Chalfont, UK). Cultures were then harvested and incorporated radioactivity was counted on a Betaplate counter (LKB Wallac, Turku, Finland).

### Generation of peptide-specific CD8^+^ T lymphocytes from healthy donors

PBMC from HLA-A2.1-positive healthy donors were cultured in complete medium at 10^6^ cells ml^−1^ concentration in the presence of antigenic or control peptides (10 *μ*g ml^−1^) encapsulated into SSL or in solution. After 7 days of culture, cells were supplemented with rIL-2 at 20 U ml^−1^ concentration. On day 10, cultures were restimulated with autologous irradiated EBV-transformed lymphoblastoid cell lines (EBV-LCL) in the presence of the above listed materials and rIL-2 at the same concentrations as above. Restimulations were performed on a weekly basis, whereas rIL-2 (20 U ml^−1^) was added to the cultures twice per week.

### Generation of tumour-infiltrating lymphocytes from melanoma biopsies

Surgical biopsies were obtained upon informed consent from melanoma patients undergoing staging procedures and single-cell suspensions were mechanically obtained. Cells were cultured in complete AB medium supplemented with rIL-2 at 200 IU ml^−1^ in 24-well culture plates previously precoated with anti-CD3 (courtesy of Dr Lanzavecchia, Bellinzona, Switzerland) and anti-CD28 (Becton Dickinson, San Jose, CA, USA) monoclonal antibodies (1 *μ*g ml^−1^). After a 2-weeks culture, over 95% of cells were CD3^+^ lymphocytes (Spagnoli *et al*, 1995).

### CTL precursor frequency

Magnetic beads (Miltenyi Biotech, Bergisch Gladbach, Germany) purified CD8^+^ TIL were resuspended in complete AB medium and seeded in 96 round-bottom wells plates (one plate for each S- or SSL-encapsulated antigen concentration) at different cell numbers (30.000, 15.000, 7.500 per well, 28 replicates for each). Autologous EBV-LCL were pulsed for 3 h with 20, 2 or 0.2 *μ*g ml^−1^ concentrations of S- or SSL-encapsulated M26–35, together with 5 *μ*g ml^−1^ *β*2-microglobulin (Sigma, St Louis, MO, USA), irradiated and added to specific limiting dilution analysis (LDA) plates at 5000 cells well^−1^. rIL-2 was added on days 4,5,6 (20 U ml^−1^ final concentration). On day 8, 2 *μ*g ml^−1^ of peptide in the same form used on day 1 was added together with rIL-2 (100 U ml^−1^ final concentration). rIL-2 was again added on day 11 at 100 U ml^−1^ final concentration. On day 15, each well was split into two and cytotoxic activity was tested by using as target cells Na-8 pulsed with M26–35 or a control peptide. Culture wells displaying ⩾25% specific cytotoxicity were considered positive and CTL precursor frequency was calculated as previously described (Chaux *et al*, 1998; Oertli *et al*, 2002).

### CTL tetramer staining

TIL from bulk cultures stimulated exactly according to the LDA protocol were stained on day 15 with HLA-A0201 tetramer-PE complex including M27–35 (Proimmune, Oxford, UK) and anti-human CD8-FITC (Becton Dickinson, San Jose, CA, USA). After 45 min incubation on ice, cells were washed with PBS and analysed by flow cytometry. Data were reported as percentages of tetramer-positive CD8^+^ T cells and mean fluorescence intensities (MFI). Positivity thresholds were established at MFI levels 10-fold higher than in unstained cells (Lee *et al*, 1999).

## RESULTS

### Construction of SSL antigenic preparations: size and epitope content stability

About 20% of the initial peptide input (19.2±6.5%) was encapsulated in SSL in the different batches used throughout this work. The stability of SSL in terms of size and peptide content was assessed at 1, 3 and 6 months after synthesis. Upon repeated photon correlation spectroscopy, different batches of SSL stored at +4°C in PBS showed nonsignificant increases in their mean diameter at 6 months only (105 *vs* 85 nm mean diameter). Peptide content remained quantitatively stable within the indicated time frame.

### Encapsulation of M27–35 peptide into SSL improves immunorecognition by specific cytotoxic T cells

The capacity of SSL-based antigenic preparations to sensitise target cells to the killing by HLA-A2.1-restricted cytotoxic T cells recognising both M26–35 and M27–35 was first tested in comparison with the corresponding soluble peptides. NA-8, HLA-A2.1^+^ melanoma cells not expressing the antigen under investigation were preincubated in the presence of graded amounts of target or control peptides in soluble form or included in SSL. Maximal killing by specific CTL was detectable upon target preincubation in the presence of 0.4 *μ*g ml^−1^ of soluble M27–35, but significantly lower (0.016–0.001 *μ*g ml^−1^) concentrations of M26–35. Encapsulation into SSL did not modulate the targeting capacity of M26–35. In contrast, inclusion of M27–35 in SSL resulted in a significant improvement of the targeting capacity of the synthetic epitope, with maximal killing being observed at 0.08 *μ*g ml^−1^ nominal peptide concentration.

### Encapsulation into SSL protects M27–35 from the action of plasma peptidases

Epitope stability in the presence of human serum or plasma has previously been investigated (Brinckerhoff *et al*, 1999; Blanchet *et al*, 2001). We asked whether encapsulation into SSL could protect peptides from plasma enzyme degradation, as indirectly measured by comparing their capacity to sensitise target cells to the killing by specific CTL. In agreement with previous reports (Blanchet *et al*, 2001), M27–35 peptide was rapidly degraded (80% loss of activity) within 45 min of preincubation in the presence of human plasma. Encapsulation of M27–35 into SSL appeared to effectively protect the epitope from enzymatic degradation, with loss of activity in those same conditions limited to 30%. On the other hand, M26–35, either in solution or included into SSL, appeared to be more resistant to hydrolysis with no loss of activity (<5%) being detectable upon 45 min incubation in the presence of plasma.

### DC as presenters of antigens encapsulated in SSL

Taken together, the above data clearly indicated that encapsulation into SSL improved immunorecognition of M27–35, possibly by protecting the synthetic epitope from enzymatic degradation. By contrast, no evident advantage appeared to be provided by M26–35 inclusion into SSL.

However, while T-cell receptor-mediated immunorecognition represents an obvious prerequisite for the induction of specific immune responses, the efficacy of vaccination procedures is critically dependent on the expansion of specific T cells. In particular, antigen-stimulated T-cell proliferation not only requires adequate costimulation, but also an increased density of MHC/peptide complexes on APC, as compared to mere targeting of cytotoxic activity (Schild *et al*, 1990; Gervois *et al*, 1996).

**Figure 1:**
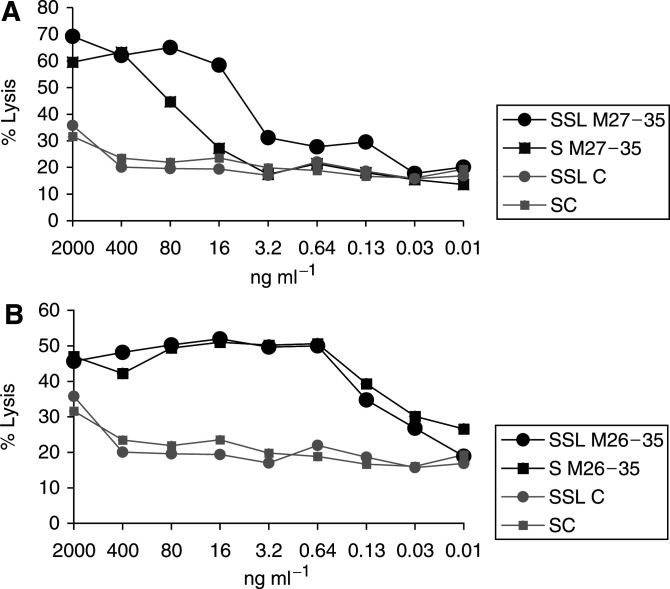
Immunorecognition of Melan-A/MART-1 epitopes by specific CTL. HLA-A2.1-positive, Melan-A/MART-1-negative NA-8 cells were 51Cr labelled and incubated for 2 h in the presence of graded amounts of Melan-A/MART-1 27–35 (M27–35, panel (**A**)) or 26–35 (M26–35, panel (**B**)) epitopes or control peptides in soluble form (SC) or encapsulated into sterically stabilised liposomes (SSL C). Cells were then washed and cultured for 4 h in the presence of a specific HLA-A2.1-restricted cytotoxic T-cell clone at 5 : 1 effector target ratio. Supernatants were then harvested and 51Cr release was measured by a gamma counter. Data are reported as mean % specific target cell lysis from triplicate wells. Standard deviations, never exceeding 10% of the reported values, were omitted.

Immunogenic class I restricted peptides are usually injected intradermally for vaccination purposes (Jaeger *et al*, 1996; Rosenberg *et al*, 1998; Marchand *et al*, 1999). The main APC type in this tissue is represented by Langerhans cells (LC), iDC that, upon antigen uptake and maturation traffick to lymph nodes, are able to activate both naive and memory T cells (Bell *et al*, 1999; Lanzavecchia and Sallusto, 2001) (Figures 1

, 2

**Figure 2:**
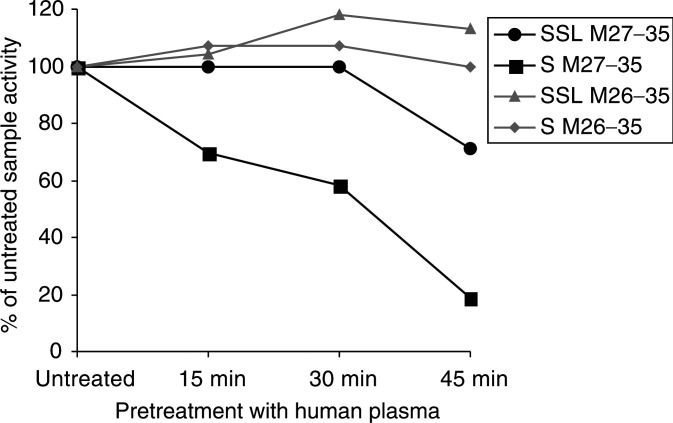
Antigenic peptide degradation in the presence of human plasma. Melan-A/MART-1 27–35 (M27–35, 1 *μ*g ml^−1^) or 26–35 (M26–35, 8 ng ml^−1^) peptides in soluble form (S) or encapsulated into SSL were incubated for the indicated times at 37°C in the presence of undiluted human plasma. The different preparations were then used to pulse 51Cr-labelled Na-8 cells for 2 h. These were cocultured for 4 h in the presence of a specific HLA-A2.1-restricted cytotoxic T-cell clone at 5 : 1 effector target ratio in triplicate samples. Supernatants were then harvested and 51Cr release was measured by a gamma counter. Data are reported as % of the cytotoxic activity detected in the presence of the corresponding untreated M27–35 or 26–35 preparations. Standard deviations, never exceeding 10% of the reported values, were omitted.

).

A convenient ‘*in vitro*’ model to explore the immunobiology of LC is represented by iDC (Romani and Schuler, 1992) obtained by culturing CD14^+^ PBMC in the presence of IL-4 and GM-CSF (Sallusto and Lanzavecchia, 1994).

**Figure 3:**
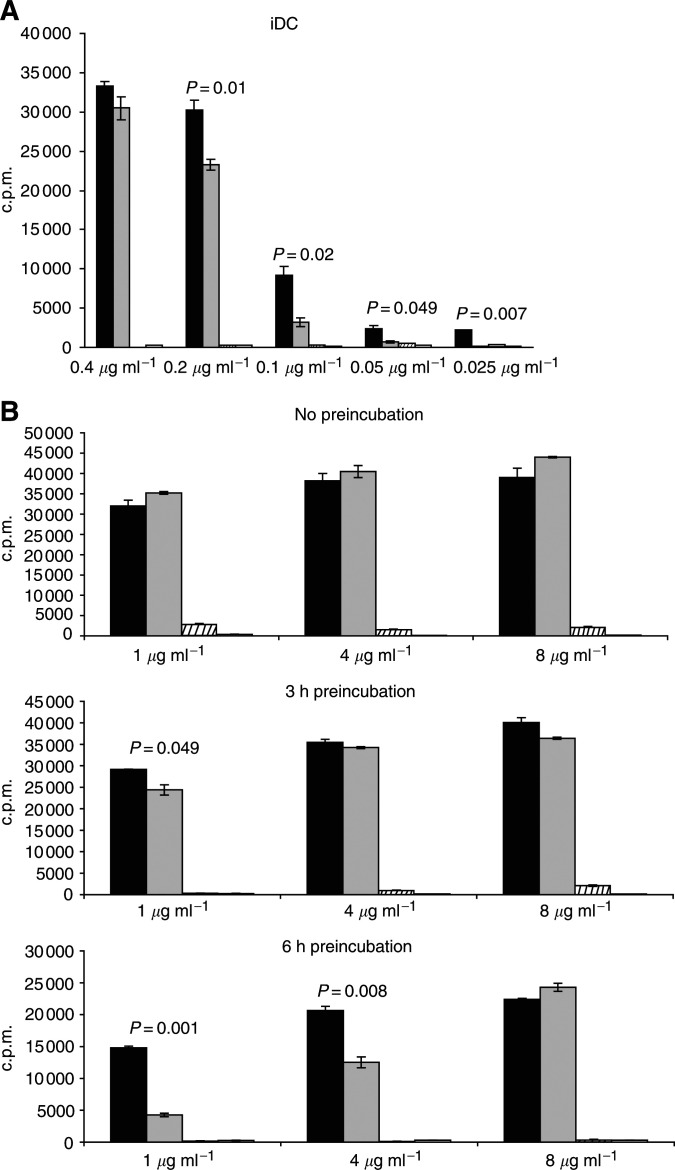
Induction of specific CTL proliferation by Melan-A/MART-1 26–35 peptide: iDC as antigen-presenting cells. (**A**) Cells from a Melan-A/MART-1-specific, HLA-A2.1-restricted CTL clone were cultured in the presence of irradiated, HLA-A2.1-matched, CD14^−^/CD1a^+^ immature, monocyte-derived dendritic cells, and the indicated concentrations of Melan-A/MART-1 26–35 or control peptides in soluble form (shaded and white columns, respectively) or contained into SSL (black and striped columns, respectively). Proliferation of triplicate wells was assessed by 3H-thymidine on day three of culture. (**B**) HLA-A2.1-positive monocyte-derived CD14^−^/CD1a^+^ iDC were cultured for 3 or 6 h (middle and lower panels, respectively) in the presence of Melan-A/MART-1 26–35 or control peptides in soluble form (shaded and white columns, respectively) or contained into SSL (black and striped columns, respectively) at the indicated concentrations. T cells from a specific CTL clone were then added. Data reported in the upper panel refer to cultures where iDC, CTL and immunogenic materials were simultaneously added without preincubation steps. In all cases, 3H-thymidine incorporation of triplicate wells was measured after 3 days culture.

HLA-A2.1 iDC were used as APC to present M26–35 in either SSL-encapsulated or soluble form to specific CTL clones. When antigenic preparations were added simultaneously together with iDC to responding T cells, inclusion into SSL determined a significant enhancement of M27–35 epitope presentation, as compared to soluble peptide (Figure 3A

). This effect was mainly observed at low nominal epitope concentrations.

Time-course experiment revealed the adverse effect of the preincubation of either antigenic preparations in the presence of iDC on the presentation to CTL, as detectable by proliferation (Figure 3B). Epitope degradation was conspicuously detectable at a 1 *μ*g ml^−1^ nominal antigen concentration. Remarkably, regarding peptides in solution, at 6 h a >80% inhibition was observed, as compared to 50% for reagents contained into SSL. Similar results were obtained when CD14^+^ monocytes were used as APC (data not shown).

These effects could be partially circumvented by increases in antigen concentrations. Thus, when the epitopes were administered at 8 *μ*g ml^−1^, either preparation induced identical CTL proliferation, with 3H-thymidine incorporation induced upon a 6 h preincubation ranging around 50% of values detectable in the presence of untreated antigen formulations.

### SSL induce specific CTL in healthy donors' PBMC

**Figure 4:**
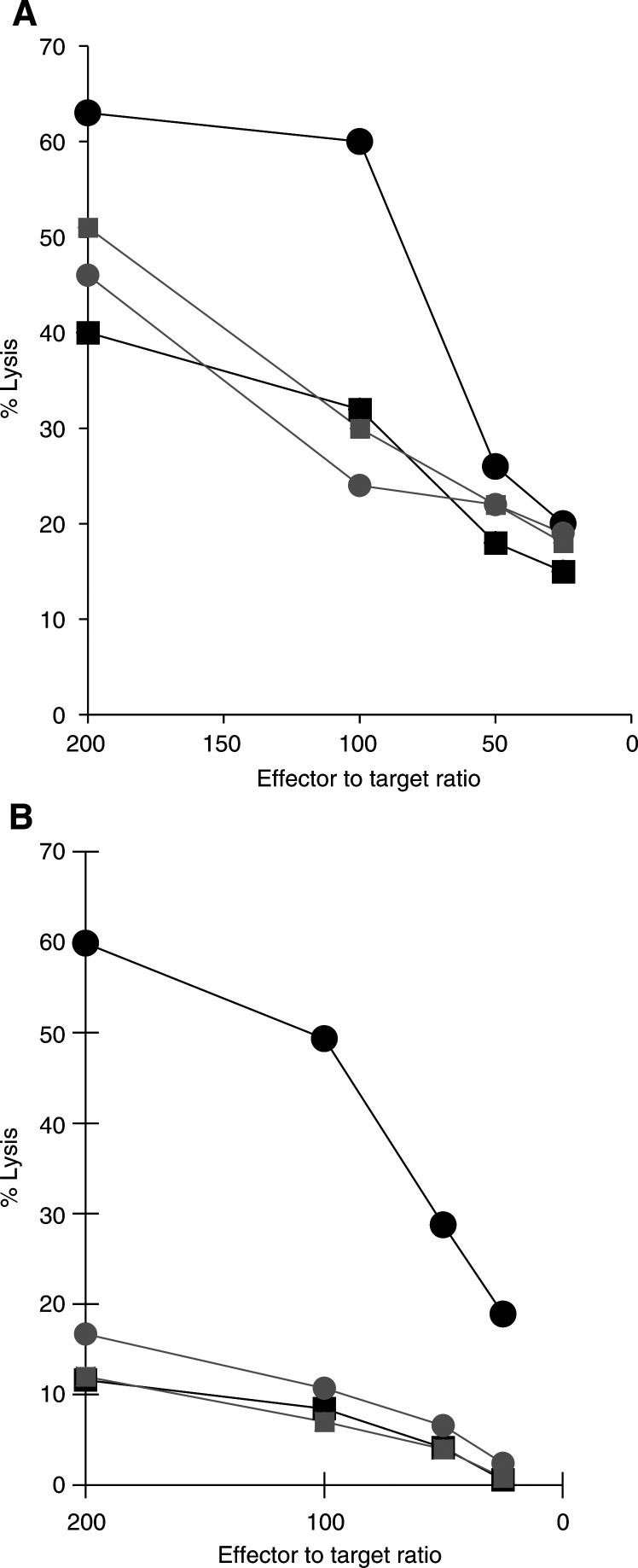
Induction of Melan-A/MART-1 specific CTL by synthetic peptides encapsulated into SSL. Healthy donor's PBMC were cultured for 1 week in the presence of Melan-A/MART-1 26–35 or control peptides at 10 *μ*g ml^−1^ in soluble form (shaded symbols) or encapsulated into SSL (black symbols). rIL-2 (20 U ml^−1^) was then added and cultures were restimulated on day 10 and weekly thereafter with the same materials in the presence of autologous irradiated EBV-LCL and rIL-2. Cytotoxic activity was tested by using as targets HLA-A2.1-positive, Melan-A/MART-1-negative cells upon pulsing with soluble M26–35 (circles) or control peptide (squares). Data from two donors (panels (**A**) and (**B**)) are shown.

The capacity of M26–35 peptide in either SSL-encapsulated or soluble form to induce specific CTL was comparatively evaluated by using PBMC from healthy donors as responder cells. In two (out of three) donors tested, following repeated (two to three) stimulations in the presence of autologous EBV-LCL, specific cytotoxic activity could indeed be generated. Remarkably, in both donors, CTL were only detectable in cultures stimulated with SSL preparations and no specific killing was observed upon soluble peptide stimulation. Figure 4

reports data from these experiments. As expectable (Oertli *et al*, 2002) M26–35-specific CTL were also able to kill HLA-A2.1^+^ melanoma cells expressing the TAA under investigation (data not shown).

### SSL induce specific CTL in melanoma derived tumour-infiltrating lymphocytes

We then comparatively tested the capacity of S or SSL M26–35 to induce specific CTL from tumour-infiltrating lymphocytes (TIL) from melanoma patients. In order to allow quantitative evaluations, we used different concentrations of immunogens and a limiting dilution analysis (LDA) setting to estimate specific CTL precursors' (CTLp) frequencies.

**Figure 5:**
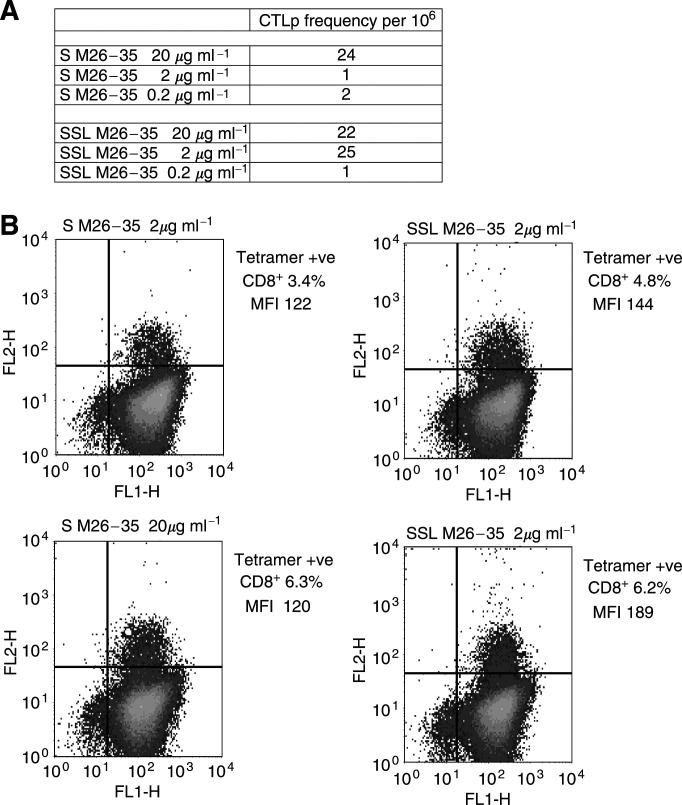
Induction of specific CTL by S M26–35 and SSL M26–35 in melanoma-derived TIL. Panel (**A**): CD8^+^ melanoma-derived TIL were stimulated in an LDA setting with decreasing amounts of S M26–35 or SSL M26–35 antigens. cytotoxic T-cells precursor frequencies are expressed as the number of specific effectors per 10^6^ CD8^+^ cells. Panel (**B**): CD8^+^ melanoma-derived TIL stimulated in bulk cultures in the presence of the indicated antigen formulations and concentrations were stained with FITC-labelled anti-CD8 and PE-labelled M27–35 tetramers, as described in ‘Materials and methods’. Percentages of tetramer-positive CD8^+^ cells and the respective MFI are reported.

Specific CTL generation was obtained by short-term culture (Oertli *et al*, 2002) in one out of two TIL preparations. At 20 *μ*g ml^−1^ antigen concentration SSL or S M26–35 peptide were equally effective, with a CTLp of about 20/10^6^. On the other hand, at 2 *μ*g ml^−1^ antigen concentration, while SSL M26–35 peptide retained its full immunogenic capacity, S M26–35 peptide was virtually unable to induce specific responsiveness. Finally, at 0.2 *μ*g ml^−1^ antigen concentration, both formulations were equally ineffective (Figure 5

).

On tetramer staining analysis, stimulation with 0.2 *μ*g ml^−1^ antigen concentration did not result in detectable expansion of tetramer-positive cells. Upon stimulation with a 2 *μ*g ml^−1^ antigen concentration of SSL M26–35, 4.8% of cells were tetramer positive, as compared to 3.4% upon S M26–35 induction (Figure 5). Finally, 20 *μ*g ml^−1^ antigen concentrations of either SSL or S M26–35 peptide induced similar percentages of tetramer-positive CD8^+^ cells (6.2 and 6.3%, respectively). Remarkably, at both antigen concentrations, MFI values were higher in cultures stimulated with SSL as compared to S M26–35 peptide (189 *vs* 120 at 20 *μ*g ml^−1^ and 144 *vs* 122 at 2 *μ*g ml^−1^).

## DISCUSSION

Following the molecular characterisation of human TAA, active specific immunotherapy is being increasingly used in cancer treatment. Critical to this purpose is the development of vaccine formulations endowed with high immunogenicity. Indeed, a large majority of human TAA are also expressed to some extent in physiological conditions (Renkvist *et al*, 2001) and immune tolerance towards them is likely to exist. Furthermore, advanced stage tumour patients frequently present ill-defined impairments of immune responsiveness. A number of reports indicate that, irrespective of discrete vaccination procedures, repeated immunisation courses are required in order to obtain detectable specific stimulation, and such responsiveness is frequently not sustained in time (Jaeger *et al*, 1996; Marchand *et al*, 1999; Thurner *et al*, 1999).

Most active specific immunotherapies currently rely on the *in vivo* administration (Jaeger *et al*, 1996; Rosenberg *et al*, 1998; Marchand *et al*, 1999) or the *ex vivo* pulsing of APC prior to re-infusion, with synthetic peptides (Nestle *et al*, 1998; Thurner *et al*, 1999). These reagents bind empty HLA molecules present in low numbers on the surface of APC. However, they are also concomitantly subject to degradation by soluble or cell membrane-associated peptidases (Amoscato *et al*, 1998; Brinckerhoff *et al*, 1999; Blanchet *et al*, 2001; Albo *et al*, 2003). As a result, they represent poor immunogens.

In this context, since persistence of the antigen (Zinkernagel and Hengartner, 2001) is emerging as a critical factor for the induction of specific immune responses, the respective bio-availabilities of different vaccine formulations assume crucial relevance.

Liposomes have long been used to carry drugs, proteins, peptides or, more recently, DNA (Alving *et al*, 1995; Lasic, 1998; Gregoriadis, 1999), and to protect them from degradation. Liposome based vaccine preparations specific for infectious agents have been successfully tested (Gluck, 1995). In particular, induction of CTL by liposome-carrying antigens has been the subject of a number of reports on animal models (Ignatius *et al*, 2000; Ludewig *et al*, 2001), but there is a conspicuous paucity of data regarding the effects in humans (Agrawal *et al*, 1998).

Based on this background, we have explored the possibility of constructing innovative vaccine formulations for clinical tumour immunotherapy, by taking advantage of well-described (Kawakami *et al*, 1994; Valmori *et al*, 1998) HLA-A2.1-restricted peptides from the immunodominant Melan-A/MART-1 melanoma TAA and liposome technology. Considering the peculiarity of tumour-specific immune response (Zinkernagel, 2001), we have focused on liposomes endowed with increased bioavailability.

Sterically stabilised liposomes are characterised by long persistence in biological fluids. As a drawback, they show reduced cellular uptake, in comparison to conventional formulations, possibly related to inhibition of the interaction with cell surfaces by the steric PEG coating (Allen, 1994).

We show here that SSL containing Melan-A/MART-1 peptides represent significantly more effective immunogenic formulations than their soluble counterparts. Regarding M27–35, their higher efficiency was already evident in immunorecognition assays by specific CTL. Furthermore, SSL could partially protect M27–35 from plasma hydrolysis.

On the other hand, M26–35 is known to be more immunogenic that M27–35 (Valmori *et al*, 1998) and more resistant to plasma peptidases (Blanchet *et al*, 2001). In this respect, SSL encapsulation did not provide any further advantage.

The effectiveness of vaccination procedures, however, critically depends on the expansion of specific T cells. Cytotoxic T-cells proliferation is characterised by more stringent molecular requirements, as compared to mere T-cell receptor-mediated target killing (Schild *et al*, 1990; Gervois *et al*, 1996). Not only costimulation is mandatory, but also higher MHC/peptide densities on APC are required.

This report clearly indicates that M26–35 encapsulation into SSL provides a significant advantage over its soluble counterpart in CTL proliferation assays, particularly when limiting amounts of antigen were used or prolonged preincubations in the presence of serum-containing medium and APC were applied.

These data are consistent with those recently reported by Ludewig *et al* (2001) in a defined mouse model, indicating that presentation of exogenous antigen to self-reactive CTL is markedly inefficient due to the rapid turnover on APC. Due to the short persistence of exogenous epitope on APC, specific CTL stimulation can only be achieved in the presence of high synthetic peptide concentrations. A major role in epitope degradation could be played by peptidases associated to APC (Amoscato *et al*, 1998; Albo *et al*, 2003).

It should be noted that the sensitivity of individual epitopes to enzymatic hydrolysis is highly variable. In this context, encapsulation of degradation-sensitive peptides into SSL is likely to provide enhanced immunogenicity.

Accordingly, M26–35 peptide encapsulated into SSL efficiently induced specific CTL in PBMC from healthy donors, following relatively few (two to three) restimulation courses. In our hands, effector cell induction could actually be induced by soluble peptides as well (Valmori *et al*, 1998; Blanchet *et al*, 2001), but usually required longer culture times (>4 restimulations).

The data obtained by using melanoma TIL as responder cells further support the concept of an enhanced immunogenicity of SSL M26–35, in comparison to S M26–35, as detected by CTLp frequency analysis and tetramer staining. In the latter assays, MFI, an indicator of functional antigen receptor avidity (Yee *et al*, 1999), was also increased in SSL M26–35-, as compared to S M26–35-stimulated cultures.

Taken together, our data suggest that SSL containing antigenic peptides could represent an effective alternative to soluble synthetic epitopes, both for direct *in vivo* administration and for *ex vivo* pulsing. Lack of intrinsic immunogenicity, favouring repeated use, low price and the possibility to easily manufacture SSL under GMP conditions concur in supporting their clinical applications.
